# Dandy-Walker Malformation and Optic Nerve Hypoplasia: A Developmental Case Highlighting the Psychiatric Impact of Central Nervous System Malformation

**DOI:** 10.7759/cureus.93718

**Published:** 2025-10-02

**Authors:** Sumeet Bhardwaj, Aayush Kapoor, Yuan Xie, Ali H Thahab, Affan Naveed, Omer Khan, Kevin Tu, Thinh D Mai

**Affiliations:** 1 Psychiatry, Kansas City University of Medicine and Biosciences, Kansas City, USA; 2 Biomedical Sciences, Edward Via College of Osteopathic Medicine, Blacksburg, USA; 3 Surgery, Kansas City University of Medicine and Biosciences, Kansas City, USA; 4 Radiology, Kansas City University of Medicine and Biosciences, Kansas City, USA

**Keywords:** autism spectrum disorder, cerebellar cognitive affective syndrome, cerebellar dysfunction, congenital brain anomaly, dandy-walker malformation, dysmetria of thought, neurodevelopmental disorders, optic nerve hypoplasia, psychiatric manifestations, sensory processing deficits

## Abstract

Dandy-Walker malformation (DWM) and optic nerve hypoplasia (ONH) are rare congenital anomalies associated with neuropsychiatric morbidity, but their co-occurrence in adults has not been described. We present an adult patient with both DWM and unilateral ONH who developed auditory hypersensitivity, visual agnosia, phonological dyslexia, dysgraphia, and longstanding academic challenges, later evolving into recurrent depression, anxiety, and insomnia. Neuroimaging revealed a Dandy-Walker variant with cerebellar atrophy and ONH, and her presentation was consistent with cerebellar cognitive affective syndrome. Notably, despite the established risk of autism spectrum-like traits in both DWM and ONH, she did not demonstrate autistic features. This case highlights how overlapping congenital anomalies may synergistically increase vulnerability to psychiatric illness and underscores the importance of early recognition and tailored interventions. To our knowledge, this is among the first adult cases describing co-occurring DWM and ONH with a longitudinal psychiatric course.

## Introduction

Literature gap

Despite the independent associations of Dandy-Walker malformation (DWM) and optic nerve hypoplasia (ONH) with psychiatric vulnerability, their co-occurrence receives minimal attention in the literature. A PubMed search (September 2025) using the terms "Dandy-Walker malformation" AND "optic nerve hypoplasia" yielded no adult cases describing longitudinal psychiatric outcomes. The term Dandy-Walker complex (DWC) encompasses DWM, DWM variants, and related posterior fossa cystic malformations. In this report, we use DWM to refer specifically to cerebellar vermis hypoplasia with cystic dilation of the fourth ventricle and posterior fossa enlargement, consistent with our patient's imaging findings. The absence of reported adult cases underscores the importance of recognizing developmental red flags early and incorporating neuropsychological tools such as the Cerebellar Cognitive Affective Syndrome/Schmahmann Scale (CCAS-S), alongside tailored psychiatric and educational interventions, to optimize long-term outcomes.

Epidemiology and risk factors

Dandy-Walker Malformation

DWM occurs in ~1 in 25-35,000 live births, with female predominance and increased familial risk [1]. Congenital or acquired cerebellar lesions are curiously observed to be associated with psychiatric disorders [2]. Because both cerebellar malformations and optic pathway anomalies have been linked to psychiatric vulnerability, ONH warrants specific attention to its distinct prenatal risk factors.

ONH has long been associated with prenatal exposures such as maternal diabetes, viral infection, alcohol, or other teratogenic agents [3]. It also often occurs in association with central nervous system abnormalities as part of a spectrum of diseases such as septo-optic dysplasia (SOD) and DWM, both congenital syndromes involving the developmental abnormalities of midline brain structures and the optic pathways [4]. While ONH is often linked to prenatal exposures, DWM more frequently reflects underlying chromosomal and genetic abnormalities.

Although most cases are sporadic, autosomal dominant and X-linked patterns are reported [1]. Chromosomal abnormalities, such as trisomies 9, 13, 18, and 21; triploidy; and 6p and 3q22-q24 deletion, are associated with DWM. Nonchromosomal syndromes such as Meckel and Walker-Warburg syndromes are also associated [5].

In a study of 187 cases of DWM, approximately one-third are reported to have a chromosomal abnormality or another genetic syndrome, the most frequent being PHACE (posterior fossa, hemangioma, arterial abnormality, cardiac malformations, eye abnormalities) syndrome; trisomy 18; and Joubert syndrome [5].

Altered nucleic acid synthesis and deficiency in granule cell precursor expansion are suspected to be key etiological factors [6]. Known risk factors include teratogen exposure (e.g., alcohol), maternal infections (e.g., cytomegalovirus (CMV), toxoplasmosis), and poorly controlled diabetes during pregnancy, all of which can disrupt cerebellar development [7].

Optic Nerve Hypoplasia

ONH, with an incidence of ~2.4 per 100,000 children, is a leading cause of congenital blindness [4]. ONH is associated with maternal malnutrition, preterm labor, and geographic clustering in socially deprived areas [8]. Milder or unilateral ONH often remains undiagnosed, and many cases are part of broader syndromes.

The etiology of ONH remains unknown [9]. ONH is described in various syndromic, chromosomal, inherited, and de novo genetic disorders. Genetic abnormalities are rare and account for only a very small fraction of cases [9].

Mutations in HESX, acting as a transcriptional repressor, are the first of a handful of genes described as causative of ONH/SOD in 1998. Other genes include mutations in PAX6, SOX2, SOX3, and OTX2 [10].

Some patients with ONH show very subtle or even absent structural abnormalities on exam or imaging, and their visual function varies widely from nearly normal vision to severe impairment. Because of this, ONH is sometimes underrecognized or harder to diagnose, depriving individuals of potentially critical interventions [3].

Psychiatric manifestations

DWM is closely associated with cerebellar cognitive affective syndrome (CCAS), which includes deficits in executive function, spatial cognition, linguistic processing, and mood regulation [11]. Key symptoms include agrammatism, distractibility, perseveration, blunted affect, and inappropriate behavior. These features overlap with attention deficit hyperactivity disorder (ADHD), obsessive-compulsive disorder (OCD), autism spectrum disorder (ASD), and bipolar disorder. However, despite these observed associations, the relationship between psychiatric symptoms and DWC is still regarded as unclear because of the lack of available data [12].

At the beginning of the 20th century, the cerebellum was regarded solely as a controller of motor functions, including diadochokinesia, tonus, coordination, and motor speech production. However, this historic view rapidly changed within the last three decades as evidence demonstrates that the cerebellum plays a key role in linguistic and cognitive development [13].

Anatomically, damage to the posterior cerebellar lobes, especially lobules VI and VII (Crus I/II), disrupts cerebellar-cortical and cerebellar-limbic connectivity, producing what Schmahmann describes as "dysmetria of thought" [11].

The CCAS-S, developed by Schmahmann et al., is a validated screening tool with higher sensitivity than the Mini-Mental State Examination (MMSE) for detecting these nonmotor cerebellar deficits [14].

Case reports link DWM to schizophrenia, bipolar disorder, and OCD. Examples include persecutory delusions, auditory hallucinations, violent behavior, and treatment resistance [12,15].

ONH is similarly associated with psychiatric and neurodevelopmental dysfunction. ASD-like behaviors are frequent in children with ONH, including social communication deficits, restricted behavior, and sensory dysregulation [16]. ASD is present in up to 25% of visually impaired children [8], with even higher rates among those with ONH [17]. One case series of 13 children with ONH reports six meeting the ASD criteria and three with partial traits [17].

## Case presentation

A 31-year-old woman with DWM and unilateral ONH presents with chronic major depressive disorder, generalized anxiety disorder, and insomnia. Since early childhood, she reports distressing auditory hypersensitivity with difficulty filtering background sounds. Environments such as classrooms, group discussions, and crowded public spaces frequently cause sensory overload characterized by irritability and headaches, consistent with auditory sensory-processing abnormalities. To cope, she often seeks quieter settings and reduced stimulation, though these strategies likely only partially mitigate symptoms and may not be consistently feasible in academic contexts.

She also describes difficulty recognizing familiar objects despite preserved single-letter identification, aligning with self-reported visual agnosia in the context of suspected cortical visual impairment related to ONH. Visually complex scenes, cluttered layouts, and rapid shifts in visual attention are particularly challenging and contribute to task avoidance and fatigue. Reading is further limited by phonological dyslexia, specifically impaired blending of letters into words despite intact letter recognition, which slows decoding and reduces reading endurance.

Written expression is constrained by impaired fine motor coordination, consistent with dysgraphia attributable to cerebellar involvement in DWM. Handwriting is less legible with speed or prolonged tasks; timed writing demands, note-taking, and form completion disproportionately tax performance compared with typed work. For this patient, these sensory, motor, and language abnormalities interfere with routine academic activities that require sustained attention in noisy settings, interpretation of visually dense material, rapid reading, or extended handwriting.

Perinatal history includes a complicated delivery attended by a midwife and inadequate prenatal care. While causality cannot be confirmed, these factors may contribute to the patient's overall neurodevelopmental profile. Despite average intellectual functioning, she experiences chronic academic underperformance and emotional dysregulation from childhood onward, with persistent anxiety around performance, episodes of sensory overstimulation characterized by difficulty tolerating auditory and visual stimuli, and sleep disruption. Over time, these lifelong sensory, cognitive, and emotional challenges culminate in adult psychiatric care focused on mood, anxiety, and insomnia.

No formal diagnosis of endocrine abnormalities is identified in the patient's documented past medical history, psychiatric records, or available medical charts. Endocrine laboratory testing (e.g., cortisol/adrenocorticotropic hormone (ACTH), thyroid-stimulating hormone (TSH)/free thyroxine (FT4), insulin-like growth factor 1 (IGF-1), gonadotropins) is not available in the records reviewed. This report is retrospective and record-based; no contemporaneous bedside neurologic testing is obtained at the time of documentation. Endocrine screening and standardized psychiatric or neuropsychological scales are likewise absent from the records and not documented in the psychiatric history provided. These limitations of the case record may have excluded clinically important information relevant to the patient's neurodevelopmental and psychiatric profile.

Serial magnetic resonance imaging (MRI)/computed tomography (CT) findings, as documented in the patient's medical records, are displayed longitudinally in Table 1 to contextualize the patient's DWM and unilateral ONH across time; the original imaging studies are not available for inclusion.

**Table 1:** Neuroimaging summary Ophthalmologic and neuroimaging findings across time. Visual acuity is expressed in Snellen notation. Years from baseline are shown to clarify the longitudinal timeline. MRI: magnetic resonance imaging; CSF: cerebrospinal fluid; DWM: Dandy-Walker malformation; w/wo = with/without; CT: computed tomography; ONH: optic nerve hypoplasia; OS: left eye; VA: visual acuity; OD: right eye

Date	Years from baseline	Modality	Findings
01/19/1999	4	MRI of the brain	Small cerebellar vermis; large posterior CSF-filled space
12/27/2000	6	MRI of the orbits/brain (w/wo contrast)	DWM variant; small bilateral optic nerves; enlarged optic sheaths
02/22/2002	7	CT of the brain (non-contrast)	Stable DWM features
09/07/2012	18	Eye exam	Nystagmus; strabismus; ONH OS; VA 20/100 OD and 6/400 OS
10/11/2023	29	CT of the head (non-contrast)	DWM findings unchanged
05/07/2024	29	MRI of the brain	DWM variant; cerebellar atrophy

**Figure 1:**
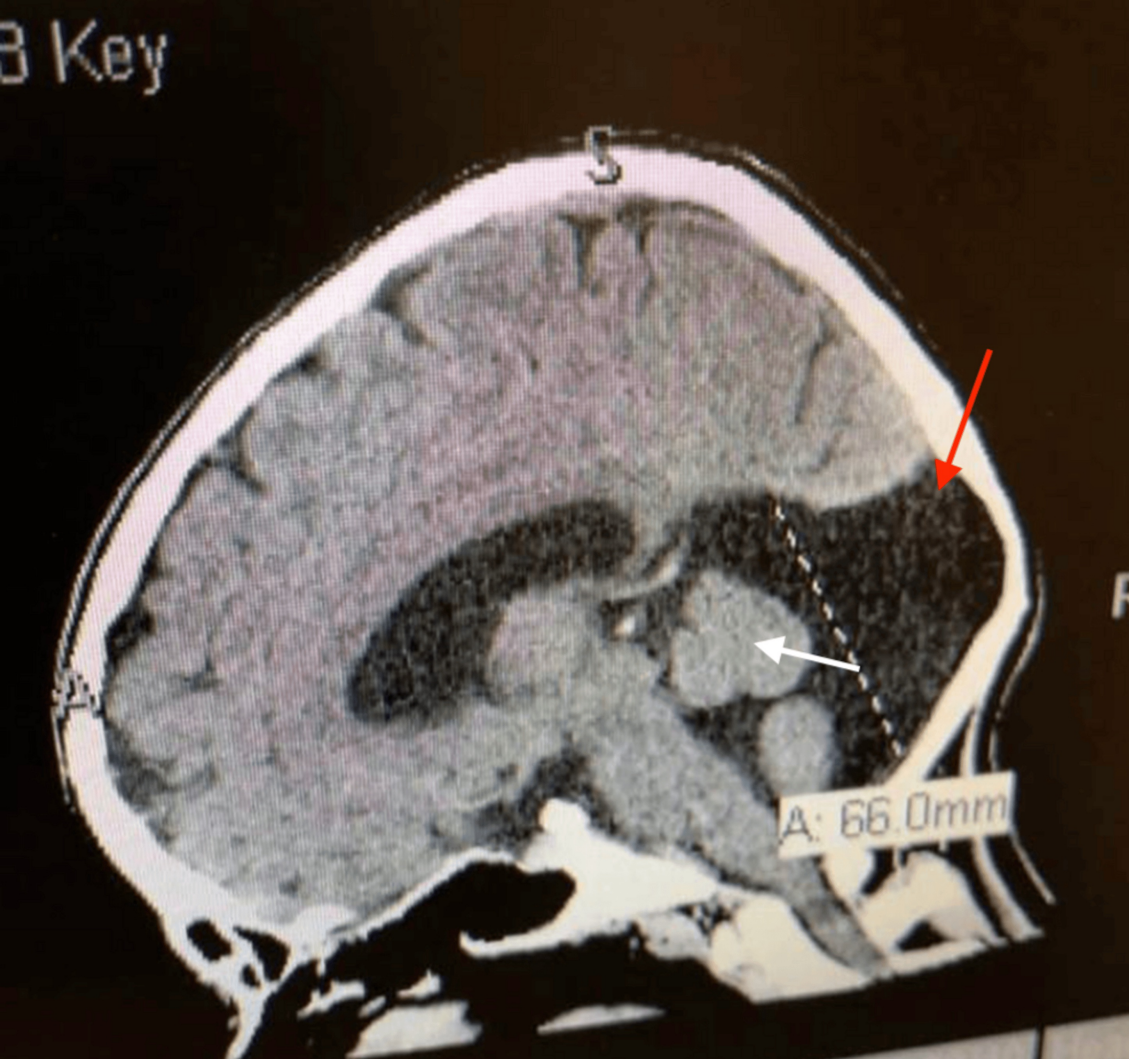
Sagittal non-contrast CT of the patient's brain demonstrating hypoplasia of the cerebellar vermis and cystic enlargement of the posterior fossa (arrows), consistent with Dandy-Walker malformation Scale bar=66 mm (11 divisions at 6 mm each) White arrow: cerebellar vermis hypoplasia; red arrow: cystic enlargement of the posterior fossa CT: computed tomography

Figure 1 depicts a non-contrast CT scan of the patient's brain demonstrating the characteristic features of DWM in the posterior fossa, providing radiographic confirmation of the diagnosis; however, the exact time of this imaging study is uncertain, as it is reported by the patient and not available in the medical record.

## Discussion

This case demonstrates how the rare co-occurrence of DWM and ONH may magnify psychiatric risk through the combined disruption of cerebellar-cognitive and sensory-limbic networks [14]. DWM is known to contribute to affective and psychotic disorders via cerebellar-limbic-prefrontal dysfunction [11,15]. ONH contributes via sensory deprivation and impaired socio-emotional development, with overlapping ASD traits [8,17]. The posterior cerebellar hemispheres, particularly lobules VI and VII (Crus I/II), form reciprocal connections with the dorsolateral prefrontal cortex, anterior cingulate, and limbic structures via the dentate nucleus and thalamus [13,18]. Lesions in these circuits impair executive control and emotional modulation, producing the hallmark features of CCAS [13]. Functional MRI studies further demonstrate reduced connectivity between the cerebellum and prefrontal regions in patients with mood and psychotic disorders, supporting the role of cerebellar dysfunction in psychiatric vulnerability [15].

ONH contributes through abnormal sensory input during critical developmental windows. Early visual deprivation reduces synaptic pruning efficiency in occipital and associative cortices, leading to compensatory reorganization that can destabilize multisensory integration and socio-emotional processing [8,16]. Endocrine abnormalities, particularly hypopituitarism, occur in a large subset of ONH patients and are underrecognized contributors to mood and cognitive symptoms [4]. Although our patient did not exhibit autistic features, she demonstrated sensory hypersensitivity and visual agnosia, supporting prior observations that ONH-related sensory disruptions may shape neurodevelopmental and psychiatric trajectories even in the absence of classic autism spectrum pathology.

Another underrecognized pathway linking DWM and ONH to psychiatric morbidity is the disruption of sleep and circadian regulation. The cerebellum participates in sleep-wake modulation through its reciprocal connections with the brainstem reticular formation and hypothalamus, and lesions in posterior lobules have been associated with fragmented sleep architecture and impaired rapid eye movement (REM) regulation [19]. Similarly, ONH frequently co-occurs with hypothalamic and pituitary dysfunction, which may impair melatonin secretion and circadian entrainment, contributing to sleep-wake rhythm disorders [20]. Chronic sleep disruption is strongly associated with heightened risk of depression, anxiety, and cognitive decline, suggesting that circadian dysregulation may represent an additional mechanism by which these anomalies potentiate psychiatric vulnerability [20].

The convergence of these anomalies, particularly in the absence of early support, may increase the risk of psychiatric illness, impaired functioning, and maladaptive coping behaviors. While each condition is studied independently in relation to mental illness, their combined impact remains underreported.

The importance of early neurodevelopmental screening in children with structural central nervous system anomalies is well established, with tools such as the CCAS-S playing a critical role in detecting cerebellar-cognitive impairment. Targeted early psychiatric and educational interventions such as structured reading programs, occupational therapy for motor-based learning difficulties, and behavioral support, together with ongoing, lifespan-oriented care coordination among neurology, psychiatry, ophthalmology, and pediatrics, are critical to minimizing preventable cognitive, emotional, and functional impairments that diminish quality of life in individuals with DWM. 

Limitations

Our report describes a single adult patient with co-existing DWM and ONH. As such, causality between these anomalies and the psychiatric manifestations cannot be decisively established, and it is difficult to generalize our findings to broader populations. Much of the patient's early developmental history is based on retrospective recall, which may be inaccurate or biased. While neuroimaging is available from childhood through adulthood, psychiatric assessments and neuropsychological testing were not consistently performed, limiting the ability to fully map the trajectory of cognitive and emotional symptoms.

Additionally, while ONH can occur as part of the SOD spectrum or other midline anomalies, there was no documentation of corpus callosum hypoplasia, pituitary abnormalities, or forebrain midline defects in this patient's records. We explicitly acknowledge that incomplete exclusion of SOD spectrum features remains a limitation due to the retrospective nature of the case and the absence of endocrine data.

The co-occurrence of DWM and ONH is extremely rare, and published literature is sparse, making direct comparison to other cases challenging. Lastly, although a potential hereditary component may play a role in this patient's health based on her records and family history, this cannot be definitively concluded at present, and further investigation is warranted.

## Conclusions

This case illustrates how rare congenital central nervous system anomalies such as DWM and ONH can synergistically produce psychiatric manifestations through complex neurodevelopmental mechanisms. Unusually, our patient demonstrates no ASD traits despite the elevated risk associated with both anomalies, highlighting the variability of psychiatric outcomes. Prior research suggests that ASD may share developmental pathways with these conditions, yet this case demonstrates that their presence does not invariably result in autism spectrum pathology. It emphasizes the importance of early identification, neuropsychological screening, and integrated multidisciplinary psychiatric care. As one of the first adult cases reported with both anomalies and a documented psychiatric trajectory, it contributes to the limited literature linking DWM with psychiatric illness and underscores the need for further research to clarify outcomes and optimize care for patients with dual central nervous system malformations. Future evaluations of patients with ONH and DWM should also include targeted endocrine testing and standardized neuropsychological assessments to better characterize potential causal contributions to psychiatric manifestations.
